# RamA, a transcriptional regulator conferring florfenicol resistance in *Leclercia adecarboxylata* R25

**DOI:** 10.1007/s12223-020-00816-2

**Published:** 2020-08-28

**Authors:** Cong Cheng, Yuanyuan Ying, Danying Zhou, Licheng Zhu, Junwan Lu, Aifang Li, Qiyu Bao, Mei Zhu

**Affiliations:** 1 Vocational and Technical College, Lishui University, Lishui, 323000 China; 2 Medical Research Center, Taizhou Hospital of Zhejiang Province, Taizhou, 317000 Zhejiang China; 3 School of Laboratory Medicine and Life Sciences/Institute of Biomedical Informatics, Wenzhou Medical University, Wenzhou, 325035 Zhejiang China; 4 The Fifth Affiliated Hospital, Wenzhou Medical University, Lishui, 323000 Zhejiang China; 5 Department of Clinical Laboratory, Zhejiang Hospital, Hangzhou, 310013 Zhejiang China

**Keywords:** *ramA*, Florfenicol, Resistance-nodulation-division, Comparative genomics, *Leclercia adecarboxylata*

## Abstract

Due to the inappropriate use of florfenicol in agricultural practice, florfenicol resistance has become increasingly serious. In this work, we studied the novel florfenicol resistance mechanism of an animal-derived *Leclercia adecarboxylata* strain R25 with high-level florfenicol resistance. A random genomic DNA library was constructed to screen the novel florfenicol resistance gene. Gene cloning, gene knockout, and complementation combined with the minimum inhibitory concentration (MIC) detection were conducted to determine the function of the resistance-related gene. Sequencing and bioinformatics methods were applied to analyze the structure of the resistance gene-related sequences. Finally, we obtained a regulatory gene of an RND (resistance-nodulation-cell division) system, *ramA*, that confers resistance to florfenicol and other antibiotics. The *ramA*-deleted variant (LA-R25Δ*ramA*) decreased the level of resistance against florfenicol and several other antibiotics, while a *ramA*-complemented strain (pUCP24-prom-*ramA/*LA-R25Δ*ramA*) restored the drug resistance. The whole-genome sequencing revealed that there were five RND efflux pump genes (*mdtABC*, *acrAB*, *acrD*, *acrEF*, and *acrAB*-like) encoded over the chromosome, and *ramA* located upstream of the *acrAB*-like genes. The results of this work suggest that *ramA* confers resistance to florfenicol and other structurally unrelated antibiotics, presumably by regulating the RND efflux pump genes in *L. adecarboxylata* R25.

**Electronic supplementary material:**

The online version of this article (10.1007/s12223-020-00816-2) contains supplementary material, which is available to authorized users.

## Introduction

Florfenicol, a derivative of chloramphenicol with better antibacterial activity and fewer adverse effects, has been widely used in veterinary medicine (Schwarz and Chaslus-Dancla 2001; Schwarz et al. 2004). However, the resistance levels and the number of resistant bacteria to florfenicol have increased due to the ever-increasing use of florfenicol in agricultural practice (Chang et al. 2015; Geng et al. 2012; Sun et al. 2016). To date, more than ten florfenicol resistance genes have been reported. These genes belong to four molecular categories: the major facilitator superfamily (MFS, including *floR*, *floRv*, *flost*, *fexA*, *fexB*, and *pexA*) (Alessiani et al. 2014; Braibant et al. 2005; He et al. 2015; Kehrenberg and Schwarz 2004; Lang et al. 2010; Liu et al. 2012); the rRNA methyltransferase family [*cfr*, *cfr*(B), and *cfr*(C)] (Hansen and Vester 2015; Schwarz et al. 2000; Tang et al. 2017); the ATP-binding cassette (ABC) family (*optrA*) (Wang et al. 2015b); and a chloramphenicol acetate esterase-encoding gene, *estDL136* (Tao et al. 2012). However, no efflux pump from RND family-related genes has been reported to be associated with florfenicol resistance.

The *ramA* global regulator belongs to the AraC/XylS family. Transcriptional regulators of the AraC/XylS family have been associated with multidrug resistance, organic solvent tolerance, oxidative stress, and virulence in Enterobacteriaceae (Gallegos et al. 1997). The *ramA* gene was first described in a multidrug-resistant (MDR) *Klebsiella pneumoniae* (George et al. 1995). Later, it was also discovered in *Enterobacter aerogenes*, *Enterobacter cloacae*, and *Salmonella enterica* (Bailey et al. 2008; Chollet et al. 2004; Keeney et al. 2007). Surprisingly, no *ramA* was identified in *Escherichia coli*. Previous studies showed that overexpression of the *ramA* gene in *E. coli* conferred decreased susceptibility to diverse antibiotics such as chloramphenicol, tetracycline, tigecycline, fluoroquinolones, and trimethoprim (Chollet et al. 2004; George et al. 1995). Concrete resistance mechanisms have been documented in *E. cloacae* and *S. enterica* serovar Typhimurium, where *ramA*-mediated overexpression of efflux pumps, primarily the AcrAB-TolC efflux pump, leads to increased tigecycline and ciprofloxacin resistance (Keeney et al. 2007; Sun et al. 2011). However, there are no available data regarding the role of the regulator gene *ramA*, which may potentially regulate the expression of other RND efflux pumps for florfenicol resistance.

*Leclercia adecarboxylata*, a motile, facultative anaerobic, Gram-negative bacillus of the Enterobacteriaceae family, is an opportunistic human pathogen that is normally present in environmental or animal sources (Stock et al. 2004; Yao et al. 2011). In this work, we used the random cloning approach to investigate the unidentified florfenicol resistance mechanism of a *L. adecarboxylata* strain R25 with the high florfenicol resistance level isolated from a rabbit anal feces sample.

## Material and methods

### The bacteria and plasmids

*L. adecarboxylata* R25 was isolated from an anal fecal sample of a rabbit during a survey of florfenicol-resistant bacteria from animal farms in 2015 in Wenzhou, Zhejiang Province, China, and it has been deposited in China Center for Type Culture Collection (CCTCC), Wuhan, China (CCTCC AB 2020046). The anal fecal samples were directly streaked on Luria-Bertani (LB) agar supplemented with 16 μg/mL florfenicol and cultured overnight at 37 °C. Bacterial identification was performed using a Vitek-60 microorganism autoanalysis system (BioMerieux Corporate, Craponne, France). The strain was further confirmed by the analysis of its 16S rRNA gene and a whole-genome sequence comparison of *L. adecarboxylata* R25 against the nucleotide database at NCBI (https://blast.ncbi.nlm.nih.gov/Blast.cgi). The bacterial strains and plasmids used in this study are listed in Table 1.

**Table 1:** Bacterial strains and plasmids used in this study

Strains and plasmids	Description	Source
Strains		
*L. adecarboxylata* R25	Multi-resistant isolate, Kan^s^, Amp^s^	This study
LA-R25Δ*ramA*	Derivative of *L. adecarboxylata* R25 that lacks the *ramA* gene	This study
pUCP24-prom-*ramA/*LA-R25Δ*ramA*	Derivative of LA-R25Δ*ramA* with wild-type *L. adecarboxylata* R25 *ramA* gene	This study
*E. coli* DH5α	Used as a host for the PCR product clone	Our lab collection
*E. coli* ATCC25922	Used as a control strain	Our lab collection
pUCP24/*E. coli* DH5α	*E. coli* DH5α carrying cloning vector pUCP24, Gm^r^	Our lab collection
pUCP24-prom-*ramA*/*E. coli* DH5α	*E. coli* DH5α carrying cloning vector pUCP24-*ramA*, Gm^r^	This study
pUCP24-prom-*omp*/*E. coli* DH5α	*E. coli* DH5α carrying cloning vector pUCP24-*omp*, Gm^r^	This study
pUCP24-prom-*mbl*/*E. coli* DH5α	*E. coli* DH5α carrying cloning vector pUCP24-*mbl*, Gm^r^	This study
Plasmids		
pKD46	Red recombinase expression, Amp^r^	Our lab collection
pCP20	FLP recombinase expression, Amp^r^	Our lab collection
pKD4	Plasmid containing an FRT-flanked kanamycin cassette, Kan^r^	Our lab collection
pUC118	Digested with *BamH*I and treated with alkaline phosphatase, Amp^r^	This study
pUCP24	Broad-host-range cloning vector, Gm^r^	Our lab collection
pUCP24-prom-*ramA*	*ramA* gene with the native promoter from *L. adecarboxylata* R25 was cloned into pUCP24, Gm^r^	This study
pUCP24-prom-*omp*	Outer membrane protease gene with the native promoter from *L. adecarboxylata* R25 was cloned into pUCP24, Gm^r^	This study
pUCP24-prom-*mbl*	MBL-fold metallohydrolase gene with the native promoter from *L. adecarboxylata* R25 was cloned into pUCP24, Gm^r^	This study

^r^Resistance; ^s^sensitive; *Amp* ampicillin, *Gm* gentamicin, *Kan* kanamycin

### Whole-genome sequencing

Bacterial genomic DNA was extracted using an AxyPrep Bacterial Genomic DNA Miniprep Kit (Axygen Scientific, Union City, CA, USA). A 20-kb library was sequenced by PacBio RS II platform (Pacific Biosciences, Menlo Park, CA) and an Illumina library with 300-bp insert sizes sequenced from both ends was obtained with a HiSeq 2500 platform (both PacBio RS II and HiSeq 2500 sequencing were finished at Annoroad Gene Technology Co., Ltd, Beijing, China). The PacBio long reads were assembled using the Canu software (Koren et al. 2017) and the assembly quality was corrected with the Illumina short reads. Potential ORFs were predicted using Glimmer software (http://ccb.jhu.edu/software/glimmer) and annotated against a nonredundant protein database using the BLASTX program (https://blast.ncbi.nlm.nih.gov). The 16S rRNA sequences were annotated by the online tool RNAmmer (http://www.cbs.dtu.dk/services/RNAmmer/).

### Random genomic DNA library construction and screening for potential florfenicol resistance genes

The genomic DNA of *L. adecarboxylata* R25 was extracted and partially digested with the *Sau3*AI enzyme. Fragments approximately 2 to 5 kb in size were retrieved from the gel and ligated into a pUC118 vector digested with *Bam*HI. The ligated sample was transformed into competent *E. coli* DH5α cells, and the transformants containing the cloned fragments were then selected on LB agar plates containing ampicillin (100 μg/mL) and florfenicol (32 μg/mL). Plasmid DNA from the positive transformant was purified, and the recombinant plasmid was subjected to PCR for the detection of the known florfenicol resistance gene (*floR*) of *L. adecarboxylata* R25 (*floR*-F: 5′-ATGACCACCACACGCCCCGC-3′ and *floR*-R: 5′-TTAGACGACTGGCGACTTCT-3′). The inserted fragments of recombinants without a known florfenicol resistance gene were sequenced, and the ORF of the potential resistance gene was obtained.

### Cloning of candidate resistance genes

Primers with restriction endonuclease adapters at both ends were used to clone the candidate gene with its potential upstream promoter region (Table 2). PrimeStarHS DNA Polymerase (TaKaRa, Dalian, China) was used to amplify the potential resistance genes according to the manufacturer’s instructions. The purified PCR product (prom-ORF) was digested with its corresponding restriction endonucleases and cloned into a pUCP24 vector that had been treated with the same restriction endonucleases. The resulting recombinant plasmid (pUCP24-prom-ORF) was transformed into *E. coli* DH5α using the calcium chloride method. The transformant was selected on LB agar plates containing 20 μg/mL gentamicin. The cloned PCR product was further confirmed by Sanger sequencing (ABI 3730 Analyzer, Foster City, CA, USA).

**Table 2:** Primers used in this work

Primer	Sequence (5′-3′)	Purpose	Product length (bp)	Annealing temperature (°n)
27F	AGAGTTTGATCCTGGCTCAG	16S rRNA	1465	55
1492R	TACGGCTACCTTGTTACGACTT			
*ramA*-knockout-F	TGCGCGTGGTGGGAATAATGTTAAATCGAAGAGCAGAGGGGGAGAGCAGCGTGTAGGCTGGAGCTGCTTC	*ramA*-knockout	1596	58
*ramA*-knockout-R	CTAAAATAGTGGCCTTAAATATCCCCTCCCGCACGGGAGGGGAATGCGCAATGGGAATTAGCCATGGTCC			
*ramA*-inner-F	TGCGCGTGGTGGGAATAATGTTAA	Inactivation identification	203	55
*ramA*-inner-R	CTAAAATAGTGGCCTTAAATATCC			
LA-*ramA*-prom-F	CGGAATTCGCTGATCAAGCTGTCGCAGGCCAGC (*EcoR*I)	*ramA* cloning	492	52
LA-*ramA*-prom-R	GCTCTAGATTAGTGCGCCCGGCTGTGGTTATCC (*Xba*I)			
LA-*mbl*-prom-F	GGGGTACCGCGGGCGATTAACGCGGTAGAAGCC (*Kpn*I)	*mbl* cloning	1398	53
LA-*mbl*-prom-R	GCTCTAGATTATTCCCACCACGCGCAAAATTGT (*Xba*I)			
LA-*omp*-prom-F	CGGAATTCTAATCTCAGCGATCCGCCGTTTCTC (*EcoR*I)	*omp* cloning	1189	52
LA-*omp*-prom-R	GCTCTAGATTAGAAACGATACTGCAGGCCGACG (*Xba*I)			

### Construction of *ramA-*knockout and *ramA-*complemented strains

The inactivation of *ramA* in the wild-type strain *L. adecarboxylata* R25 was performed according to the method described by Datsenko and Wanner (Datsenko and Wanner 2000). Briefly, a kanamycin resistance gene (*aph*) flanked by FLP recognition target (FRT) sites in pKD4 was amplified by PCR using *ramA*-knockout-F/R primers and the template plasmid pKD4 under standard conditions. The *ramA*-knockout-F/R primers (Table 2) consisted of 20 nucleotides (nt) of the helper plasmid pKD4 and 50 nt of the 5′ and 3′ ends of the corresponding inactivated gene (*ramA*). The purified PCR fragment was digested with *Dpn*I, purified, and transformed into *L. adecarboxylata* R25 by electroporation in the presence of pKD46 (carrying the Red recombinase gene). The mutant strain (LA-R25Δ*ramA*-*aph*) was verified by PCR using the *ramA*-inner-F/R primers. The *aph* gene was further excised from LA-R25Δ*ramA*-*aph* by the plasmid pCP20, which encodes FLP nuclease, introduced via transformation. Finally, the *ramA-*deleted variant was obtained and named LA-R25Δ*ramA*.

The recombinant plasmid with *ramA* and its upstream predicted promoter region (pUCP24-prom-*ramA*) was transformed into the *ramA*-deleted strain (LA-R25Δ*ramA*), and the transformant (pUCP24-prom-*ramA/*LA-R25Δ*ramA*) was selected on LB agar containing 20 μg/mL gentamicin. The plasmid (pUCP24-prom-*ramA*) in the transformant (pUCP24-prom-*ramA/*LA-R25Δ*ramA*) was confirmed by PCR with primers targeting *ramA* and further sequenced by Sanger sequencing.

### Antibiotic susceptibility assay

The MICs of the antimicrobial agents against *L. adecarboxylata* R25 and other strains, including LA-R25Δ*ramA*, pUCP24-prom-*ramA*/LA-R25Δ*ramA*, and pUCP24-prom-*ramA*/*E. coli* DH5α, were determined by the standard agar dilution method recommended by CLSI-2017 (the Clinical and Laboratory Standards Institute in 2017). The measurements of MICs in the presence of the carbonyl cyanide m-chlorophenylhydrazone (CCCP) were also carried out by standard agar dilution method containing serial dilutions of six antibiotics (florfenicol, tetracycline, erythromycin, nalidixic acid, clarithromycin, and levofloxacin) in 18 mL of Mueller-Hinton broth (MHB), followed by the addition of 2 ml of CCCP (100 μg/mL in dimethyl sulfoxide [DMSO]) to give a final concentration of 10 μg/mL. The MIC was recognized as the lowest antibiotic concentration showing no colony growth. Each of the tests was carried out in triplicate. *E. coli* ATCC 25922 was used as a quality control strain.

### qRT-PCR analysis of *ramA* mRNA concentration in *L. adecarboxylata* R25 and the recombinant strains

To validate the transcription level of the *ramA* gene in vivo, qRT-PCR was performed using the StepOne™ RT-PCR System (Applied Biosystems, USA) (Sanchez-Abarca et al. 2010). Total RNAs were extracted from the wild, mutant, and recombinant strains cultured in LB with or without florfenicol using the Trizol. cDNA was obtained by reverse transcription using the PrimeScript RT-PCR Kit (TaKaRa, Dalian, China). After qPCR analysis, relative quantification of the target in each sample was calculated using rpsL as the internal control (Mikhail et al. 2019).

### Bioinformatic analysis of the genetic environment of *ramA*

Sequences containing the *ramA* gene were obtained from the NCBI nucleotide database by the BLAST program using an approximately 10-kb fragment (including the sequences of the upstream 1-kb region and the downstream 9-kb region of the *ramA* gene) of the *L. adecarboxylata* R25 genome sequence as the query. A total of 100 complete bacterial genome sequences sharing the greatest sequence identity with the 10-kb fragment of the *L. adecarboxylata* R25 genome were retrieved (until March 15th, 2018). The sequences were filtered to accept only those encoding at least one of the four genes (*tetR*, *ramA*, and *acrA*-, and *acrB*-like) with an amino acid identity ≥ 50%. The sequences were clustered into different groups according to the number and order of the four homologous genes. A representative sequence in each group was chosen as the candidate for structural comparative analysis.

## Results

### The antibiotics susceptibility of *L. adecarboxylata* R25 and resistance genes encoded in the *L. adecarboxylata* R25 genome

*L. adecarboxylata* R25 showed high resistance levels to florfenicol and chloramphenicol, with both MIC levels of 128 μg/mL. The strain also showed high resistance levels to several other antibiotics, such as tetracycline (MIC 256 μg/mL) and linezolid (MIC 128 μg/mL) (Table 3). The whole genome of *L. adecarboxylata* R25 (CP035382.1) consists of a circular chromosome (4.74 Mb) and two plasmids, pLA64 (64 kb, CP035381.1) and pLA109 (109 kb, CP035380.1). The functional annotation revealed that the whole genome encoded 9 drug resistance genes (*mdfA*, *floR*, *aac*(*6′*)-*Ib*-*cr*, *arr-3*, *dfrA27*, *aadA16*, *qacE*Δ*1*, *sul1*, and *qnrB6*). Of the 9 resistance genes, only one resistance gene *floR* was reported conferring resistance to florfenicol.

**Table 3:** MICs of antibiotics for the *Leclercia adecarboxylata* R25 strain and its derivatives (μg/mL)

Strain	FFN	CHL	LZD	TET	ERY	TGC	NAL	MY	RIF	AMP	GEN	STR	AMK	KAN
*L. adecarboxylata* R25	128	128	128	256	16	1	8	512	256	2	< 0.03	4	8	4
LA-R25Δ*ramA*	64	64	64	128	8	1	4	256	256	2	< 0.03	4	8	4
pUCP24-prom-*ramA/*LA-R25Δ*ramA*	1024	1024	1024	16	128	> 4	16	> 1024	128	2	–	4	4	4
pUCP24-prom-*ramA*/*E. coli* DH5α	64	64	> 1024	16	256	2	16	> 1024	16	2	–	4	8	1
pUCP24-prom-*omp*/*E. coli* DH5α	8	8	–	–	–	–	–	–	–	–	–	–	–	–
pUCP24-prom-*mbl*/*E. coli* DH5α	8	8	–	–	–	–	–	–	–	–	–	–	–	–
pUCP24/*E. coli* DH5α	8	8	256	2	64	0.25	4	512	16	2	–	4	8	1
*E. coli* DH5α	8	8	256	2	64	0.25	4	512	16	2	0.06	4	8	1
*E. coli* ATCC25922	2	2	128	2	32	0.25	< 2	512	16	2	0.06	4	16	1

*FFC* florfenicol, *CHL* chloramphenicol, *LZD* linezolid, *TET* tetracycline, *ERY* erythromycin, *TGC* tigecycline, *NAL* nalidixic acid, *MY*, lincomycin, *RIF* rifampin, *AMP* ampicillin, *GEN* gentamicin, *STR* streptomycin, *AMK* amikacin, *KAN* kanamycin

### A randomly cloned *ramA* gene in *E. coli* conferring florfenicol resistance

A transformant with florfenicol and chloramphenicol resistance and free of the known florfenicol resistance gene was obtained from random cloning of R25 genomic DNA. The 2.8-kb insert fragment encoded three ORFs, including an MBL-fold metallohydrolase (*mbl*), a RamA family antibiotic efflux transcriptional regulator (*ramA*), and an outer membrane protease (*omp*), which shared 96, 100, and 54% amino acid identity with the top hits in the UniProtKB database, A0A2S4X220, A0A2T3CIN1, and A0A078LS26, respectively, of which the gene of RamA (A0A2T3CIN1) was from *Enterobacter* sp. FS01 isolated from parkland soil, Aarhus, Denmark. The minimum inhibitory concentration (MIC) results for the cloned genes with the predicted promotor regions (pUCP24-prom-*ramA*/DH5α, pUCP24-prom-*omp*/DH5α, pUCP24-prom*-mbl*/DH5α) showed that only the recombinant with *ramA* (pUCP24-prom-*ramA*/DH5α) was responsible for multidrug resistance, including resistance to florfenicol and chloramphenicol, and had the same MIC levels as the original transformant with the 2.8-kb insert fragment. The other two recombinants (pUCP24-prom-*omp*/DH5α and pUCP24-prom-*mbl*/DH5α) did not show any resistance to the antibiotics (Table 3).

### Prominent functions of *ramA* in mediating antimicrobial resistance in *L. adecarboxylata* R25 and its derivatives

The recombinant strain pUCP24-prom-*ramA*/DH5α exhibited increased MIC levels against florfenicol (MIC increased from 64 to 1024 μg/mL, 8-fold), chloramphenicol (8-fold), linezolid (> 4-fold), tetracycline (8-fold), erythromycin (4-fold), tigecycline (8-fold), nalidixic acid (4-fold), and lincomycin (> 2-fold) relative to recipient *E. coli* DH5α or *E. coli* DH5α carrying the vector pUCP24 (pUCP24/DH5α) (Table 3). No differences in rifampicin, ampicillin, streptomycin, amikacin, and kanamycin resistance were observed among these strains. LA-R25Δ*ramA* showed 2-fold decreased resistance levels against florfenicol and chloramphenicol relative to the parental strain *L. adecarboxylata* R25. After complementation with *ramA* (pUCP24-prom-*ramA*), the resistance levels of the recombinant (pUCP24-pro-*ramA/*LA-R25Δ*ramA*) to florfenicol and chloramphenicol were fully recovered, with even higher resistance levels than the wild-type *L. adecarboxylata* R25 (MIC increased from 64 to 1024 μg/mL, 16-fold). Similar results were also found for the other antibiotics (Table 3). To further evaluate the role of *ramA* in the resistance of the strains to florfenicol, the transcription levels of *ramA* and its potential targets in *L. adecarboxylata* R25 were determined. It revealed that the wild strain *L. adecarboxylata* R25 cultured with florfenicol showed significantly higher transcription level of *ramA* than the one cultured without it. The *ramA*-complemented LA-R25Δ*ramA* (pUCP24-pro-*ramA*/LA-R25Δ*ramA*) which showed much higher MIC level to florfenicol (1024 μg/mL) than that (128 μg/mL) of the wild strain *L. adecarboxylata* R25 also showed significantly higher transcription level of *ramA* than that of the wild strain *L. adecarboxylata* R25 (Table 4). The expression levels of the potential target genes of *ramA* lowered in the *ramA*-deleted *L. adecarboxylata* R25 (LA-R25Δ*ramA*) and recovered in *ramA*-complemented strain (pUCP24-pro-*ramA*/LA-R25Δ*ramA*). They had (much) higher expression levels when the wild strain *L. adecarboxylata* R25 was treated with florfenicol (Table 4). It seemed that they were all regulated by *ramA* even though at different degrees.

**Table 4:** qRT-PCR results of the transcription of *rmaA* in *Leclercia adecarboxylata* R25 and its derivatives

	*ramA*	*mdtB*	*mdtC*	*acrD*	*mdtF*	*acrB*	*acrB-like*
*L. adecarboxylata* R25	1	1	1	1	1	1	1
*L. adecarboxylata* R25 + F^a^	2192.19	194.09	5.65	2.99	2.41	20.95	5.52
LA-R25Δ*ramA*	0.2	1.33	0.48	0.35	0.34	0.71	0.78
pUCP24-pro-*ramA*/LA-R25Δ*ramA*	1805.52	48.9	1.86	1.29	1.15	2.79	2.64

^a^The strain cultured with florfenicol (64 μg/mL)

To confirm if the florfenicol resistance of *L. adecarboxylata* R25 was related with the efflux pumps, CCCP was used as an inhibitory agent for the efflux pumps. CCCP itself exhibited an antimicrobial effect on *L. adecarboxylata* R25 and its derivatives with MICs of 20 μg/mL. We tested the MIC levels of the antibiotics combined with CCCP at a lower concentration (10 μg/mL). It showed that CCCP increased the sensitivity of *L. adecarboxylata* R25 and LA-R25Δ*ramA* against florfenicol, tetracycline, and levofloxacin to 4- to 8-fold, but it did not show any effect on erythromycin, clarithromycin, and nalidixic acid (Table 5).

**Table 5:** MIC levels of *Leclercia adecarboxylata* R25 and its derivatives to the antibiotics in the presence or absence of CCCP (μg/mL)^a^

	FFC		TET		ERY		CLR		NAL		LEV	
	− I	+ I	− I	+I	− I	+I	− I	+I	− I	+I	− I	+ I
*L. adecarboxylata* R25	128	16	128	32	32	16	8	8	8	8	0.125	0.016
LA-R25Δ*ramA*	64	16	128	16	8	8	4	8	4	2	0.032	< 0.008
pUCP24-prom-*ramA/*LA-R25Δ*ramA*	1024	512	8	8	32	32	32	16	16	16	0.125	0.125

^a^The bacterial strains were treated with (+ I) or without (− I) 10 μg/mL CCCP

*FFC* florfenicol, *TET* tetracycline, *ERY* erythromycin, *CLR* clarithromycin, *NAL* nalidixic acid, *LEV* levofloxacin

### Structural comparison of the genetic environment of *ramA* and five RND systems encoded on the *L. adecarboxylata* R25 chromosome

**Fig. 1:**
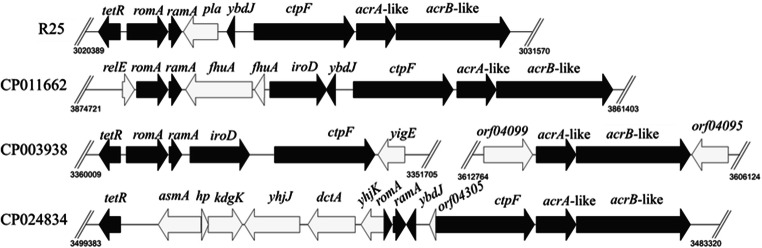
Comparison of the gene organization of *ramA* and its genetic environment in *L. adecarboxylata* R25 with corresponding regions in other representative strains. Regions encoding the same or different genes are indicated by black or white arrows, respectively

Based on their similarity to the four ORFs (encoded by *ramA* and the other three genes in its neighboring region) in *L. adecarboxylata* R25, 96 sequences were chosen for further homology analysis. All were from chromosomes of species belonging to the family Enterobacteriaceae and were clustered into four groups. Four representative sequences from each group are illustrated in Fig. 1 and Table S1. Structural analysis of the four representatives showed that three of them contained a TetR family transcriptional regulator, which was absent in CP011662. Interestingly, the four representatives all contained two RND family efflux transporter genes, *acrA*-like and *acrB*-like. Moreover, a set of genes, including *asmA*, *hp*, *kdgK*, *yhjJ*, *dctA*, and *yhjK* were inserted between *tetR* and *ramA* in CP024834.

**Fig. 2:**
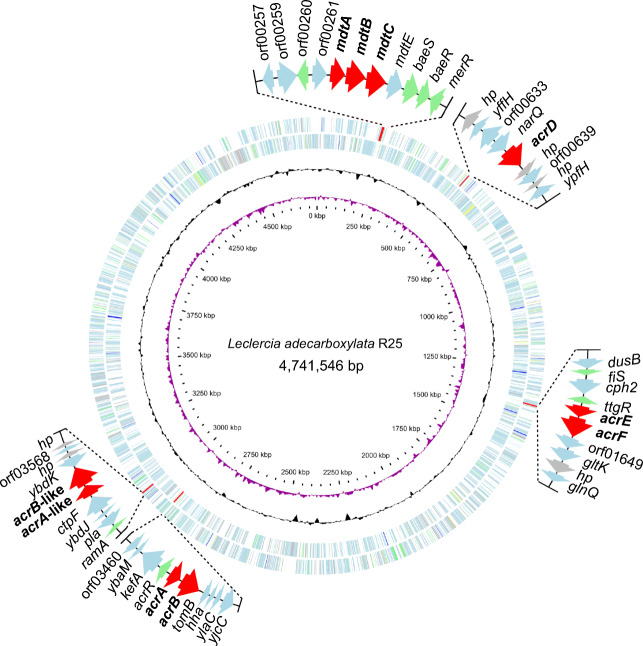
Genome map of *L. adecarboxylata* R25. The circles, from the inner to the outside: (1) the position in kb. (2) GC skew (G − C/G + C), with a positive GC skew toward the outside and a negative GC skew toward the inside. (3) GC content, with an average of 50%, and a G + C content of more than 50% is shown toward the outside; otherwise, inward. (4) Genes encoded in the leading strand (outwards) or the lagging strand (inwards). Genes with different functions are shown in different colors, and five groups of RND efflux pump-related elements are in red.

The chromosome of *L. adecarboxylata* R25 encoded four clusters of antibiotic resistance-related IMP-MFP genes and an IMP gene independent of the RND systems (Table 6 and Fig. 2). These genes included *mdtABC*, *acrEF*, *acrAB*, *acrAB*-like, and *acrD*. In the neighboring regions of the four clusters of IMP-MFP genes, the predicted regulators of RND systems were identified. These regulators were *ramA*, *baeSR*, *ttgR*, and *acrR*, respectively, of which only two (*ramA* and *baeSR*) were positive regulators (activating the transcription of the RND-type efflux pumps) (Fig. 2).

**Table 6:** The RND efflux pump genes identified in the *L. adecarboxylata* R25 genome

IMP or MFP gene	Annotation	Size (bp)	Gene name	AA identity (%)^a^
ORF00264	Multidrug transporter subunit MdtA	1233	*mdtA*	80.96
ORF00265	Multidrug transporter subunit MdtB	3126	*mdtB*	90.11
ORF00267	Multidrug transporter subunit MdtC	3078	*mdtC*	92.49
ORF00637	Aminoglycoside/multidrug transporter subunit AcrD	3114	*acrD*	92.86
ORF01643	Efflux RND transporter periplasmic adaptor subunit	1140	*acrE*	73.77
ORF01647	Multidrug efflux RND transporter permease subunit	3114	*acrF*	83.51
ORF03455	MexE family multidrug efflux RND transporter periplasmic adaptor	1194	*acrA*	77.83
ORF03454	Aminoglycoside/multidrug transporter permease	3141	*acrB*	92.37
ORF03562	Efflux RND transporter periplasmic adaptor subunit	1065	*acrA*-like	27.56
ORF03564	Hydrophobe/amphiphile efflux-1 family RND transporter	3096	*acrB*-like	38.67

^a^The amino acid sequence identities of the membrane fusion protein or inner membrane protein genes of the RND systems in *L. adecarboxylata* R25 with *mdtA*, *mdtB*, *mdtC*, *acrD*, *acrE*, *acrF*, *acrA*, *acrB*, *acrA*, and *acrB* of *E. coli* DH5α (CP026085.1), respectively

## Discussion

It has been reported that *ramA* is associated with tigecycline and fluoroquinolone resistance, but its association with resistance to florfenicol has not been described. In this work, however, we found that a *ramA* gene encoded on the chromosome of *L. adecarboxylata* R25 was involved in resistance to florfenicol and several other antibiotics. This result was in accordance with previous observations that the *ramA* gene in *S. enterica* serovar Typhimurium or *K. pneumoniae* was associated with resistance to tigecycline and other antibiotics. Deletion of *ramA* resulted in increased susceptibility to antibiotics, and complementation with plasmid-borne *ramA* restored the parental phenotype of antimicrobial resistance (Horiyama et al. 2011; Wang et al. 2015a). These results confirmed that *ramA* played a role in bacterial resistance to florfenicol as well as other antibiotics.

The RND efflux pump system mainly consists of an inner membrane transporter protein such as AcrB, a periplasmic MFP such as AcrA, and an outer membrane channel protein such as TolC (Nikaido and biology 2011). All IMPs usually exist as trimers, and their external loops contain binding sites for ligands, while their transmembrane domains mainly function as a conduit for protons that serve as the energy source. During the export of substrates, IMPs undergo three consecutive conformational changes, including moving through access, binding, and extrusion conformations. In addition, with its broad substrate specificity, IMPs play their most important role in the RND efflux pump system. Previous studies have demonstrated that *ramA* is a regulatory factor involved in the upregulation of the expression of AcrAB and its paralogs, which mediate bacterial resistance to many chemical agents, including antibiotics. *ramA* in *S. enterica* serovar Typhimurium increases resistance to ciprofloxacin and other antimicrobial agents via upregulation of AcrAB expression (Sun et al. 2011). A similar study also showed that *ramA* upregulates the expression of AcrEF, a paralog of AcrAB, in *S. enterica* serovar Typhimurium (Bailey et al. 2010). A similar result was reported by Sheng et al., who demonstrated that a high *ramA* expression level in *K. pneumonia* was simultaneously accompanied by high expression of *acrB*, which conferred high resistance to tigecycline (Sheng et al. 2014). On the other hand, *ramA*-inactivated *K. pneumoniae* KP17 downregulated *acrB*, which decreased tigecycline resistance (Wang et al. 2015a). In this work, in accordance with these previous reports, the expression levels of the potential IMP genes (*mdtB*, *mdtC*, *acrD*, *mdtF*, *acrB*, and *acrB-like*) decreased in the *ramA*-deleted *L. adecarboxylata* R25 (LA-R25Δ*ramA*) and recovered in *ramA*-complemented strain (pUCP24-pro-*ramA*/LA-R25Δ*ramA*). These results demonstrated that RamA might activate the expression of some IMPs and enhance the antibiotic resistance of bacteria.

## Electronic supplementary material

ESM 1 (DOC 104 kb)
