# Supplementary material for: RamA, a transcriptional regulator conferring florfenicol resistance in Leclercia adecarboxylata R25
Source: Folia Microbiol (Praha). 2020 Aug 28;65(6):1051–60. doi: 10.1007/s12223-020-00816-2 (PMC7716942; doi:10.1007/s12223-020-00816-2)
Supplement: Supplementary file 1 — (DOC 104 kb) [file 12223_2020_816_MOESM1_ESM.doc]

Table S1 The *ramA-*harboring sequences collected from the NCBI Nucleotide database

| Accession number | Group | Bacteria | Location |
| --- | --- | --- | --- |
| CP035382.1 | 1 | *Leclercia adecarboxylata* R25 | chromosome |
| CP012165.1 | 1 | *Enterobacter hormaechei* subsp. oharae strain 34978 | chromosome |
| CP021896.1 | 1 | *Enterobacter cloacae* strain AR_0050 | chromosome |
| CP017180.1 | 1 | *Enterobacter hormaechei* subsp. oharae strain DSM 16687 | chromosome |
| CP027142.1 | 1 | *Enterobacter hormaechei* subsp. hoffmannii strain AR_0365 | chromosome |
| CP022148.1 | 1 | *Enterobacter cloacae* strain 704SK10 | chromosome |
| CP019839.1 | 1 | *Enterobacter cloacae* strain R11 | chromosome |
| CP009850.1 | 1 | *Enterobacter cloacae* strain ECNIH4 | chromosome |
| CP001918.1 | 1 | *Enterobacter cloacae* subsp. cloacae ATCC 13047 | chromosome |
| CP017475.1 | 1 | *Enterobacter cloacae* strain M12X01451 | chromosome |
| CP016906.1 | 1 | *Enterobacter cloacae* isolate SBP-8 | chromosome |
| CP017179.1 | 1 | *Enterobacter hormaechei* subsp. steigerwaltii strain DSM 16691 | chromosome |
| CP010384.1 | 1 | *Enterobacter hormaechei* subsp. oharae strain 34399 | chromosome |
| CP011591.1 | 1 | *Enterobacter asburiae* strain CAV1043 | chromosome |
| CP024812.1 | 1 | *Enterobacter* sp. CRENT-193 | chromosome |
| CP017183.1 | 1 | *Enterobacter xiangfangensis* strain LMG27195 | chromosome |
| CP021902.1 | 1 | *Enterobacter cloacae* strain AR_0136 | chromosome |
| CP021776.1 | 1 | *Enterobacter cloacae* strain AR_0053 | chromosome |
| CP018814.1 | 1 | *Enterobacter cloaca*e strain AR_0002 | chromosome |
| CP012167.1 | 1 | *Enterobacter hormaechei* subsp. steigerwaltii strain 34998 | chromosome |
| CP025034.1 | 1 | *Lelliottia nimipressuralis* strain SGAir0187 | chromosome |
| CP017186.1 | 1 | *Enterobacter hormaechei* subsp. hoffmannii strain DSM 14563 | chromosome |
| CP003678.1 | 1 | *Enterobacter cloacae* subsp. dissolvens SDM | chromosome |
| CP007546.1 | 1 | *Enterobacter asburiae* L1 | chromosome |
| CP019889.1 | 1 | *Enterobacter cloacae* strain FRM | chromosome |
| CP021137.1 | 1 | *Enterobacter* sp. DKU_NT_01 | chromosome |
| LT840187.1 | 1 | *Enterobacter cloacae* strain DG6 | chromosome |
| CP010376.2 | 1 | *Enterobacter hormaechei* subsp. steigerwaltii strain 34977 | chromosome |
| CP026719.1 | 1 | *Enterobacter cloacae* strain AR_0060 | chromosome |
| CP014280.1 | 1 | *Enterobacter cloacae* isolate MBRL1077 | chromosome |
| CP021749.1 | 1 | *Enterobacter cloacae* strain AR_0163 | chromosome |
| CP020053.1 | 1 | *Enterobacter cloacae* strain AR_0065 | chromosome |
| CP022532.1 | 1 | *Enterobacter cloacae* strain MS7884A | chromosome |
| CP009756.1 | 1 | *Enterobacter cloacae* strain GGT036 | chromosome |
| CP002886.1 | 1 | *Enterobacter cloacae* EcWSU1 | chromosome |
| CP018785.1 | 1 | *Enterobacter cloacae* strain AA4 | chromosome |
| CP006580.1 | 1 | *Enterobacter cloacae* P101 | chromosome |
| CP003026.1 | 1 | *Enterobacter asburiae* LF7a | chromosome |
| CP015774.2 | 1 | *Lelliottia amnigena* strain ZB04 | chromosome |
| CP023529.1 | 1 | *Lelliottia amnigena* strain FDAARGOS_395 | chromosome |
| CP025963.2 | 1 | *Klebsiella pneumoniae* strain WCHKP34 | chromosome |
| CP014071.1 | 1 | *Klebsiella quasipneumoniae* strain ATCC 700603 | chromosome |
| CP014156.1 | 1 | *Klebsiella quasipneumoniae* strain HKUOPL4 | chromosome |
| CP014155.1 | 1 | *Klebsiella quasipneumoniae* strain HKUOPJ4 | chromosome |
| CP014696.2 | 1 | *Klebsiella quasipneumoniae* strain ATCC 700603 | chromosome |
| CP014154.1 | 1 | *Klebsiella quasipneumoniae* strain HKUOPA4 | chromosome |
| CP012300.1 | 1 | *Klebsiella pneumoniae* subsp. pneumoniae strain HKUOPLC | chromosome |
| CP012252.1 | 1 | *Klebsiella variicola* strain HKUOPLA | chromosome |
| CP010512.1 | 1 | *Enterobacter cloacae* strain colR/S | chromosome |
| CP026850.1 | 1 | *Enterobacter cloacae* strain AR_0072 | chromosome |
| CP025225.1 | 1 | *Enterobacter cancerogenus* strain CR-Eb1 | chromosome |
| CP026387.1 | 1 | *Leclercia* sp. LSNIH3 | chromosome |
| CP013990.1 | 1 | *Leclercia adecarboxylata* strain USDA-ARS-USMARC-60222 | chromosome |
| CP024838.1 | 1 | *Klebsiella pneumoniae* strain CRKP-1215 | chromosome |
| CP023925.1 | 1 | *Klebsiella pneumoniae* strain FDAARGOS_442 | chromosome |
| CP021939.1 | 1 | *Klebsiella pneumoniae* strain AR_0145 | chromosome |
| CP021944.1 | 1 | *Klebsiella pneumoniae* strain AR_0152 | chromosome |
| CP021757.1 | 1 | *Klebsiella pneumoniae* strain AR_0138 | chromosome |
| CP017985.1 | 1 | *Klebsiella pneumoniae* strain 825795-1 | chromosome |
| CP018719.1 | 1 | *Klebsiella pneumoniae* strain KP_Goe_828304 | chromosome |
| CP018713.1 | 1 | *Klebsiella pneumoniae* strain Kp_Goe_152021 | chromosome |
| CP018707.1 | 1 | *Klebsiella pneumoniae* strain Kp_Goe_827026 | chromosome |
| CP018701.1 | 1 | *Klebsiella pneumoniae* strain Kp_Goe_827024 | chromosome |
| CP018695.1 | 1 | *Klebsiella pneumoniae* strain Kp_Goe_149832 | chromosome |
| CP020061.1 | 1 | *Klebsiella pneumoniae* strain AR_0117 | chromosome |
| CP017087.1 | 1 | *Enterobacter* sp. HK169 | chromosome |
| CP020817.1 | 1 | *Enterobacter* sp. Crenshaw | chromosome |
| CP012999.1 | 1 | *Enterobacter* sp. E20 | chromosome |
| CP010377.1 | 1 | *Enterobacter hormaechei* subsp. hormaechei strain 34983 | chromosome |
| CP011863.1 | 1 | *Enterobacter asburiae* strain ATCC 35953 | chromosome |
| CP017990.1 | 1 | *Enterobacter cloacae* complex sp. ECNIH7 | chromosome |
| CP009854.1 | 1 | *Enterobacter cloacae* strain ECNIH5 | chromosome |
| CP008905.1 | 1 | *Enterobacter cloacae* ECR091 | chromosome |
| CP008897.1 | 1 | *Enterobacter cloacae* ECNIH3 | chromosome |
| CP017184.1 | 1 | *Enterobacter roggenkampii* strain DSM 16690 | chromosome |
| CP012162.1 | 1 | *Enterobacter cloacae* complex sp. 35734 | chromosome |
| CP003737.1 | 1 | *Enterobacter cloacae* subsp. cloacae ENHKU01 | chromosome |
| CP015227.1 | 1 | *Enterobacter* sp. ODB01 | chromosome |
| CP017279.1 | 1 | *Enterobacter ludwigii* strain EN-119 | chromosome |
| CP011798.1 | 1 | *Enterobacter cloacae* strain UW5 | chromosome |
| CP000653.1 | 1 | *Enterobacter* sp. 638 | chromosome |
| FP929040.1 | 1 | *Enterobacter cloacae* subsp. cloacae NCTC 9394 | chromosome |
| CP011662.1 | 2 | *Enterobacter hormaechei* strain CAV1176 | chromosome |
| CP011650.1 | 2 | *Enterobacter cloacae* strain CAV1669 | chromosome |
| CP011584.1 | 2 | *Enterobacter cloacae* strain CAV1668 | chromosome |
| CP011581.1 | 2 | *Enterobacter cloacae* strain CAV1411 | chromosome |
| CP011572.1 | 2 | *Enterobacter cloacae* strain CAV1311 | chromosome |
| CP008823.1 | 2 | *Enterobacter cloacae* ECNIH2 | chromosome |
| CP026975.1 | 2 | *Enterobacter cloacae* complex sp. strain FDAARGOS_77 | chromosome |
| CP003938.1 | 3 | Enterobacteriaceae bacterium strain FGI 57 | chromosome |
| LT556085.1 | 3 | *Citrobacter* sp. 92 | chromosome |
| LT556084.1 | 3 | *Citrobacter* sp. 86 | chromosome |
| CP018628.1 | 3 | *Lelliottia jeotgali* strain PFL01 | chromosome |
| CP024834.1 | 4 | *Klebsiella pneumoniae* strain CRKP-2297 | chromosome |
| CP023913.1 | 4 | *Klebsiella pneumoniae* strain FDAARGOS_439 | chromosome |
| CP015382.1 | 4 | *Klebsiella pneumoniae* strain CN1 | chromosome |
| CP001891.1 | 4 | *Klebsiella variicola* At-22 | chromosome |
| AP018340.1 |  | *Enterobacter cloacae* subsp. strain 2016_smu953 | chromosome |
| CP026167.1 |  | *Leclercia* sp. LSNIH1 | chromosome |
| XM_011569066.1 |  | *Plutella xylostella* uncharacterized LOC105397058 | chromosome |
| CP014993.1 |  | *Enterobacter asburiae* strain ENIPBJ-CG1 | chromosome |
